# Mast Cell Proteases 6 and 7 Stimulate Angiogenesis by Inducing Endothelial Cells to Release Angiogenic Factors

**DOI:** 10.1371/journal.pone.0144081

**Published:** 2015-12-03

**Authors:** Devandir Antonio de Souza, Antonio Carlos Borges, Ana Carolina Santana, Constance Oliver, Maria Célia Jamur

**Affiliations:** Department of Cell and Molecular Biology and Pathogenic Bioagents, Ribeirão Preto Medical School, University of São Paulo, Ribeirão Preto, SP, Brazil; University of Bari Medical School, ITALY

## Abstract

Mast cell proteases are thought to be involved with tumor progression and neo-vascularization. However, their exact role is still unclear. The present study was undertaken to further elucidate the function of specific subtypes of recombinant mouse mast cell proteases (rmMCP-6 and 7) in neo-vascularization. SVEC4-10 cells were cultured on Geltrex^®^ with either rmMCP-6 or 7 and tube formation was analyzed by fluorescence microscopy and scanning electron microscopy. Additionally, the capacity of these proteases to induce the release of angiogenic factors and pro and anti-angiogenic proteins was analyzed. Both rmMCP-6 and 7 were able to stimulate tube formation. Scanning electron microscopy showed that incubation with the proteases induced SVEC4-10 cells to invade the gel matrix. However, the expression and activity of metalloproteases were not altered by incubation with the mast cell proteases. Furthermore, rmMCP-6 and rmMCP-7 were able to induce the differential release of angiogenic factors from the SVEC4-10 cells. rmMCP-7 was more efficient in stimulating tube formation and release of angiogenic factors than rmMCP-6. These results suggest that the subtypes of proteases released by mast cells may influence endothelial cells during *in vivo* neo-vascularization.

## Introduction

Mast cells are connective tissue cells that are involved in allergy, inflammation and host defense [1–5]. The location of the mast cell as well as their ability to produce and release a variety of chemical mediators is essential in the pathophysiology of allergic and inflammatory reactions [6–9]. A number of studies have functionally linked mast cells to tumor angiogenesis [10–14]. Mast cells have been shown to accumulate around several types of tumors and are generally the first inflammatory cells to infiltrate tumors [15, 16]. Preformed mast cell mediators such as heparin, histamine, TNF-α, and bFGF have been shown to stimulate the proliferation of endothelial cells [13, 17–19], thus suggesting that mast cell mediators could be important for blood vessel formation and/or maintenance [20–23]. However, some preformed mast cell mediators are also produced by other cell types such as macrophages, endothelial cells, and fibroblasts, which impedes delineation of the specific role of mast cells in angiogenesis.

The major constituents of mast cell secretory granules are the mast cell specific proteases: chymase, tryptase, and CPA3 (carboxypeptidase A3) [6, 24–29]. The majority of recent investigations on the role of mast cells in tumor angiogenesis have focused on the ability of mast cells to synthesize, store, and release mast cell specific chymases and tryptases. Several these studies have shown that tryptase can act directly or indirectly in the degradation and remodeling of the extracellular matrix during angiogenesis [30, 31]. Zhi and colleagues [32] have shown that tryptase induces cell proliferation, migration, and tube formation in mouse brain endothelial cells, suggesting a role for tryptase in microvessel formation. Furthermore, mMCP-6 (mouse mast cell protease 6) and mMCP-7 (mouse mast cell protease 7), both tryptases, were able to induce spreading and tube formation in SVEC4-10 endothelial cells [33].

The previous results noted that the tryptase subtypes have differing efficiencies in promoting spreading and tube formation, suggesting that they may have different physiological and pathological roles in angiogenesis. The present study was undertaken to further elucidate the mechanisms by which the specific subtypes of mast cell tryptases stimulate endothelial cells during angiogenesis. The current investigation confirms that rmMCP-6 and rmMCP-7 have differing effects on endothelial cells, both in their ability to induce tube formation and in their capacity to release angiogenic factors.

## Materials and Methods

### Ethics Statement

The research was conducted in accordance with Ethical principles in the use of experimental animals adopted by the Brazilian College of Animal Experimentation. Experimental protocols were approved by the Commission on Ethics on Animal Experimentation of the Ribeirão Preto Medical School (Protocol number 033/2007).

### Cell Lines

The murine endothelial cell line SVEC4-10 (CRL-2181) was purchased from the American Type Culture Collection (ATCC; Manassas, VA). The cells were maintained in Dulbecco's Modified Eagle's Medium (DMEM) plus 10% heat inactivated fetal bovine serum (FBS) according to ATCC guidelines. The cells were cultured in a humidified environment containing 5% CO_2_ in air. All reagents used for cell culture were purchased from Life Technologies (Carlsbad, CA).

### Primary Culture of Bone Marrow-derived Murine Mast Cells (BMMC)

Three young (8 to 12 weeks) male BALB/c mice were anesthetized with ketamine 80 mg/kg plus xylazine 12 mg/kg (Sigma-Aldrich, St.Louis, MO). Bone marrow was removed from the femurs and cultured according to Jamur and colleagues [34]. After 21 days in the culture, all the cells were mast cells. These mast cells were used for production of pre-formed mast cell mediators.

### Pre-formed Mast Cell Mediators

To obtain pre-formed mast cell mediators [26], BMMC cells were incubated with 0.1 μM calcium ionophore-A23187 (Sigma-Aldrich) for 45 min at 37°C and the supernatant collected and used in tube formation assays. To confirm the release of mediators, the supernatant was analyzed by western blot for mMCP-6.

### *In vitro* angiogenesis—Tube Formation Assay

10μl of Geltrex^®^ (Life Technologies) was added to each well of μ-slides Angiogenesis^®^ (IBIDI, Martinsried, Germany) and allowed to solidify at 37°C for 30 min. After the gel solidified, SVEC4-10 cells (1x10^4^) in 50μl of DMEM supplemented with 10% FBS were added to each well. The cells were incubated at 37°C in a humidified atmosphere (95% air/5% CO_2_) for 5 h in the presence or absence of rmMCP-6 or 7 (20ng/well; R&D Systems Inc., Minneapolis, MN). In some experiments, the proteases were pre-incubated using antibodies against mMCP-6 and 7 (kindly provided by Dr. Michael F. Gurish, Division of Immunology, Brigham and Women’s Hospital, Harvard Medical School, Cambridge, MA). The proteases (20ng) were incubated with the respective antibody (1μg) for 30 min before adding them to the cell culture. The antibody concentration was determined using a dose-response curve for blocking tube formation. For fluorescence experiments, the cells were fixed, permeabilized, and then incubated with 2.6 U/ml Phalloidin-Alexa 488 (Life Technologies) for 30 min [35]. Following staining, the samples were washed in PBS and observed with an inverted fluorescence microscope (Nikon Eclipse TE2000-U, Nikon instruments Inc., NY, USA) and images acquired using a Nikon DS-1QM digital camera. The images were acquired using a 10X objective and the measurements are expressed in pixels (800x600) where 1 pixel is equal to 0.069mm^2^. Tube formation was quantified using WimTube (Wimasis Image Analysis, Munich, Germany).

The parameters [36–39] used for quantification were:

Covered area (%): the area covered by cells that are part of a tubular structure.
Tubes: part of a tubular structure that go from one branching point to another branching point or to a loose end.
Branching points: the points where three or more tubes converge
Loops: enclosed (or almost enclosed) areas inside the tubular structure that fulfill roundness conditions.

### *In vivo* chick embryo chorioallantoic membrane (CAM) assay

Fifteen fertilized eggs were incubated at 37°C with 60% humidity. On day 2 of incubation 3 ml of albumen were removed using a syringe with a 21g needle. A square window was then opened in the eggshell and the window covered with Scotch^®^ Magic^™^ tape (3M, St. Paul, MN) to prevent dehydration and contamination. At day 8 of incubation, 13 mm Thermanox^™^ (Thermo Scientific^™^ Nunc^™^, Rochester, NY) rings were placed on the CAM under sterile conditions. 100 ng rmMCP-6 or 7 in 20μL of assay buffer (50 nM MES, 1M NaCl, pH 6.5) was placed into the Thermanox^™^ rings. For controls 20 μL of assay buffer only were placed in the Thermanox^™^ rings. On day 12 of incubation, the CAM was fixed *in situ* with 2% paraformaldehyde for 20 min. The Thermanox^™^ rings with the underlying CAM were cut out and transferred to a petri dish containing PBS. Samples were stained with hematoxylin and eosin. The samples were mounted on glass slides and a minimum of five fields/sample was analyzed with a 4x objective on an Olympus BX-50 microscope (Olympus America Inc., Melville, NY) equipped with a SPOT RT3 digital camera (Diagnostic Instruments, Inc., Sterling Heights, MI).

### Scanning Electron Microscopy

Geltrex^®^ was placed on 12-mm-round coverslips coated with Biobond (Electron Microscopy Sciences, Hatfield, PA). After the gel solidified, SVEC4-10 cells (1x10^4^) were added in 50μl of DMEM supplemented with 10% FBS. The cells were incubated at 37°C in a humidified atmosphere (5% CO_2_ in air) for 5 h in the presence or absence of rmMCP-6 or 7 (20ng; R&D Systems Inc.). The samples were then rinsed in warm PBS (37°C) and fixed in 2% glutaraldehyde (Ladd Research Industries; Burlington, VT) in warm PBS for 2 hours at room temperature. Samples were postfixed in 1% OsO_4_ (EM Sciences) for 2 hours, rinsed in Milli-Q water, and incubated with a saturated solution of thiocarbohydrazide (EM Sciences), followed by 1% OsO_4_. This step was repeated once. The samples were dehydrated in a graded ethanol series and critically point-dried with liquid CO_2_ in a Tousimis Autosandri-810 (Tousimis Research Co., Rockville, MD), mounted on aluminum stubs with silver paint (EM Sciences), and coated with gold in a BAL-TEC SCD 050 Sputter Coater (BAL-TEC AG). Samples were examined with a JEOL JSM-6610LV scanning electron microscope (JEOL, Ltd.; Tokyo, Japan).

### Immunoblotting and Zymography

Following the tube formation assay, the culture supernatants were used for immunoblotting to analyze metalloprotease expression and for zymograms to determine metalloprotease activity. The Geltrex^®^ on the μ-slides after the tube formation assay was used to analyze the presence of laminin and collagen IV. The attached cells were removed from the Geltrex^®^ by incubation for 20 min with TrypLE^™^ Express (Life Technologies). The Geltrex^®^ was recovered using 50μl of lysis buffer. The protein concentration of the sample (Geltrex^®^ plus lysate buffer) was determined using the BCA Protein Assay Kit (Pierce, Thermo Fisher Scientific, Rockford, IL). 10 μg of sample (Geltrex^®^ plus lysate buffer) were boiled for 5 min in 1x SDS sample buffer (50 mM Tris-HCl pH 6.8, 12.5% glycerol, 1% sodium dodecylsulfate, 0.01% bromophenol blue), and applied to 10% polyacrylamide gels. Immunoblotting was performed as previously described [33] using anti-laminin (SC16589; Abcam Cambridge, MA) and anti-collagen IV (ab6586); Santa Cruz Biotechnology, Santa Cruz, CA) antibodies. The secondary antibodies goat anti-rabbit IgG conjugated to HRP and rabbit anti-goat IgG conjugated to HRP were purchased from Jackson ImmunoResearch (West Grove, PA).

The supernatants from the tube formation assays were used for zymography. The supernatants were centrifuged at 2800 *g* at 4°C for 30 min. The protein concentration of the supernatants and lysates were determinate using the BCA Protein Assay. Quantitative gelatinolytic zymography was done using the method of Leber and Balkwill [40]. The samples were subjected to electrophoresis under nonreducing conditions on 7.5% SDS-polyacrylamide gels copolymerized with 2 g/L porcine skin gelatin (Sigma Aldrich). After electrophoresis, gels were washed in 2.5% Triton-X 100 (Sigma Aldrich) with agitation and then incubated for at least 24 hours at 37°C in enzyme incubation buffer (50 mM Tris-HCl, pH 7.5, containing 5 mM CaCl_2_, 100 mM NaCl, 0.01% Triton X-100, 0.1 mM ZnCl_2_, 0.2% Brij (Sigma Aldrich), and 0.002% NaN3). Gels were stained in 0.2% Coomassie Brilliant Blue R-250 (Sigma Aldrich) and then destained in water. The optical density of the bands was determined using Adobe Photoshop (Adobe Systems, San Jose, CA).

### Expression Profile of Angiogenesis Related Proteins

The expression profile of angiogenesis-related proteins was analyzed using the Proteome Profiler^™^ Mouse Angiogenesis Antibody Array (R&D Systems). After the tube formation assay, the supernatants were collected and processed according to the manufacturer’s instructions. Briefly, supernatants were mixed with a cocktail of biotinylated detection antibodies and then incubated with the membrane containing immobilized angiogenesis-related antibodies. Bound protein was detected with streptavidin conjugated to HRP. Membranes were washed and developed using ECL^™^ Western Blotting Detection Reagent RPN2106 (GE Healthcare, Piscataway, NJ). Supernatants from tube formation assays performed without proteases served as controls.

### Statistical Analysis

Values are expressed as the mean ± SD. Student’s t-test was used to compare data. *p*-values of ≤0.05 were considered significant. The data is expressed as mean ± SD from three independent experiments.

## Results

### Pre-formed mast cell mediators induced tube formation

**Fig 1:**
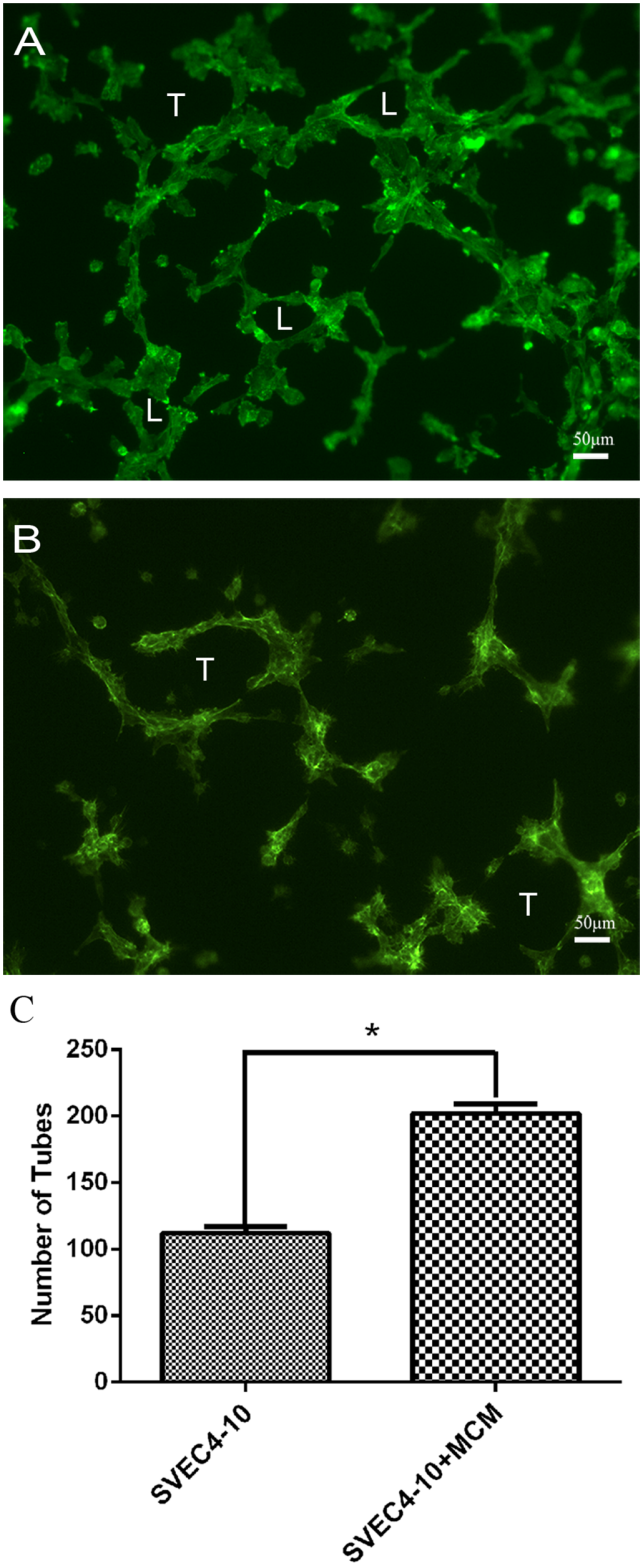
In the presence of pre-formed mast cell mediators, SVEC4-10 cells form tubes and loops. The cells were cultured for 5 hours at 37°C on Geltrex^®^ in the presence of Mast Cell Mediators (MCM) from bone marrow derived mast cell. (A) When the SVEC4-10 cells were cultured in the presence of MCM, most of the cells were spread on the substrate, and formed both tubes (T) and loops (L). (B) When the cells were incubated in absence of mast cell mediators, the cells were spread on the substrate forming tubes (T), but there was no loop formation. (C) Quantitative analysis shown that the tube formation was higher in presence of mast cell mediators. The significance was determined by Student's *t*-test *p ≤ 0.05.

In order to confirm that mast cell mediators stimulate tube formation, the ability of the released pre-formed mast cell mediators to induce tube formation was tested. Bone marrow derived mast cells were stimulated with calcium ionophore A23187 and SVEC4-10 cells cultured with the medium containing the released mast cell mediators. The mast cell mediators were highly efficient in stimulating tube and loop formation (Fig 1A) in SVEC4-10 cells when compared to the SVEC4-10 cells cultured in the absence of mast cell mediators (Fig 1B). Quantitative analysis confirmed the data obtained by fluorescence microscopy (Fig 1C), suggesting that pre-formed mast cell mediators, including tryptases, can stimulate *in vitro* angiogenesis.

### Mast cell tryptases increased tube formation

**Fig 2:**
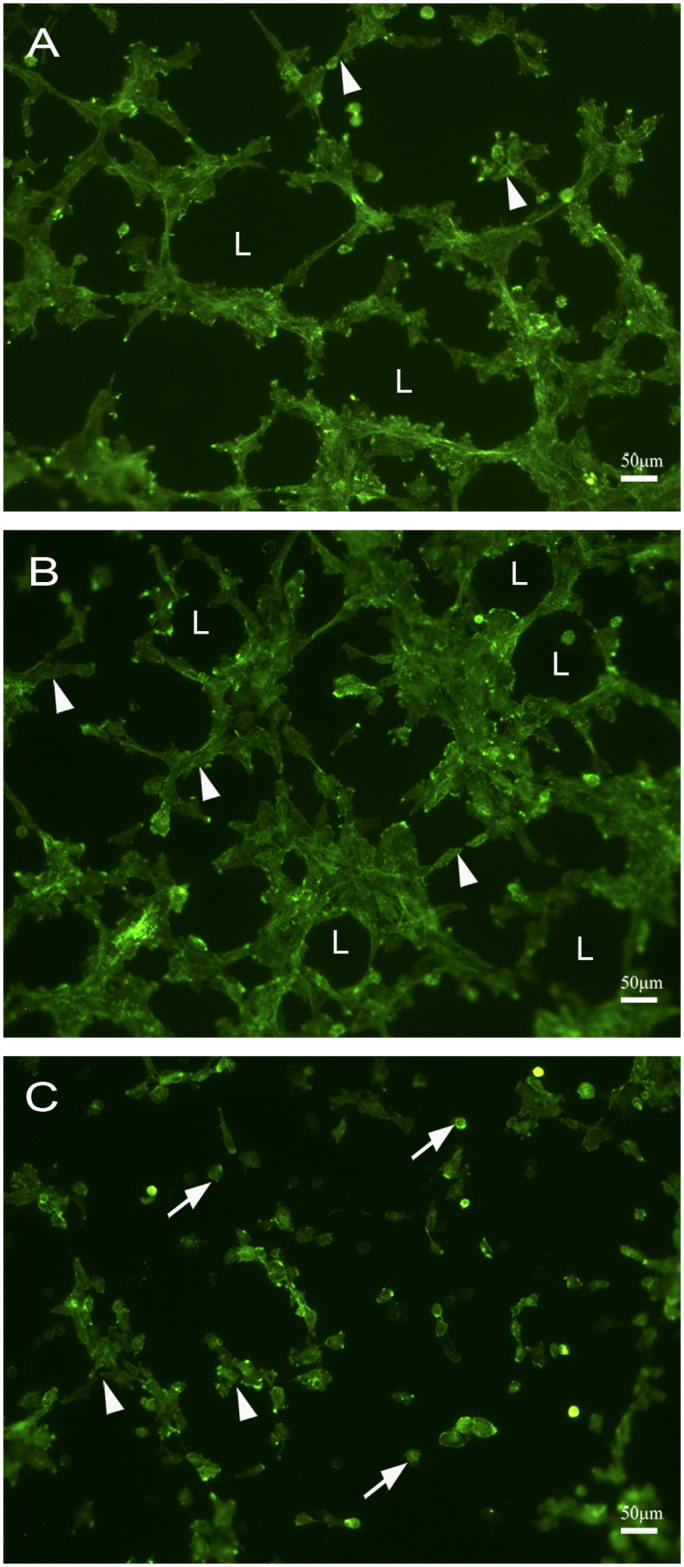
rmMCP-6 and 7 induce tube formation by endothelial cells. SVEC4-10 cells were cultured for 5 hours at 37°C on Geltrex^®^ in the presence of rmMCP-6 (A), rmMCP-7 (B) or in the absence of tryptases (C). (A) In the presence of rmMCP-6 most of the SVEC4-10 cells were spread on the substrate, tube formation was observed (arrowhead) and loops were also present (L). (B) When the SVEC4-10 cells were cultured with rmMCP-7, tubes (arrowheads) and loops (L) were more prevalent. (C) When SVEC4-10 cells are cultured in the absence of tryptases (control) only a few cells were seen in the initial phase of tube formation (arrowhead) and most of the cells remained unspread (arrow). Cells were stained with phalloidin conjugated to Alexa 488. Five independent experiments were performed.

To directly evaluate the ability of specific mast cell tryptases, rmMCP 6 and 7, to induce tube formation an *in vitro* angiogenesis assay was performed using SVEC4-10 cells. After 5 hours of incubation with rmMCP-6 most SVEC4–10 cells were spread on the substrate, and formed both tubes and loops (Fig 2A). When the cells were incubated with rmMCP-7 there was an increase in the number of tubes and loops when compared with incubation with rmMCP-6 (Fig 2B). After 5 hours of culture in the absence of proteases, only a few SVEC4–10 cells were spread on the substrate forming tubes, but there was no loop formation (Fig 2C).

**Fig 3:**
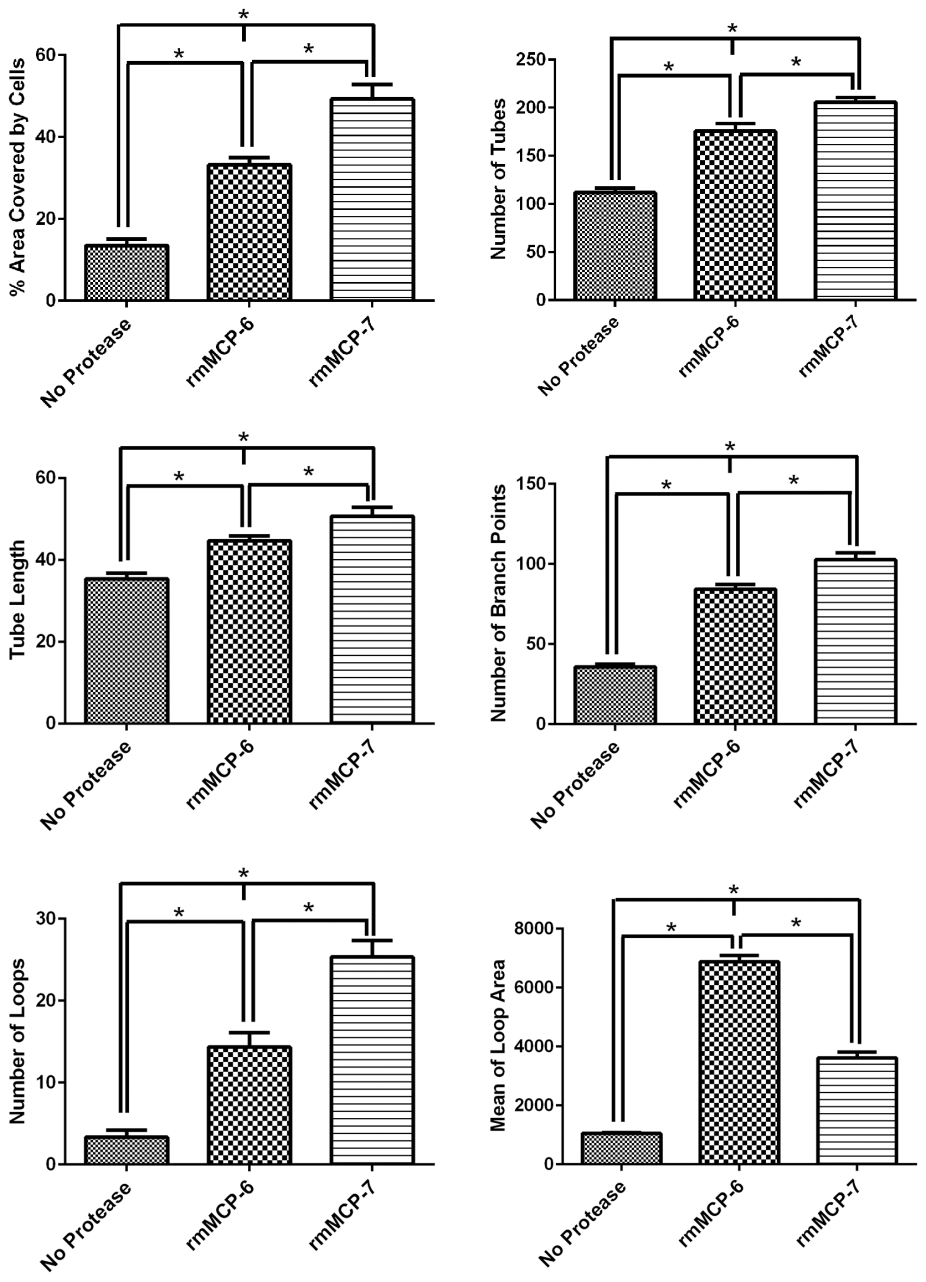
rmMCP-7 is more effective in inducing tube formation by endothelial cells *in vitro*. SVEC4-10 cells were cultured for 5 hours at 37°C on Geltrex^®^ in the presence of rmMCP-6, rmMCP-7 or in the absence of tryptases. The area covered by tubes, the tube length, number of tubes, loops and branching points, the average area occupied by the loops were quantified using Wimasis WimTube. Tube length and mean of loop area are expressed in number of pixels. Data are presented as mean ± SD from five independent experiments. *p ≤ 0.05.

A quantitative analysis confirmed the data obtained by fluorescence microscopy (Fig 3). The area covered by tubes, the number of tubes, the tube length, branches and loops were significantly higher when SVEC4-10 cells were cultured with rmMCP-7. On the other hand, the average area occupied by loops was higher when endothelial cells were cultured with rmMCP-6 in comparison to culture with rmMCP-7. To further confirm the direct action of these proteases on tube formation the proteases were pre-incubated with antibodies against either mMCP-6 or mMCP-7.

**Fig 4:**
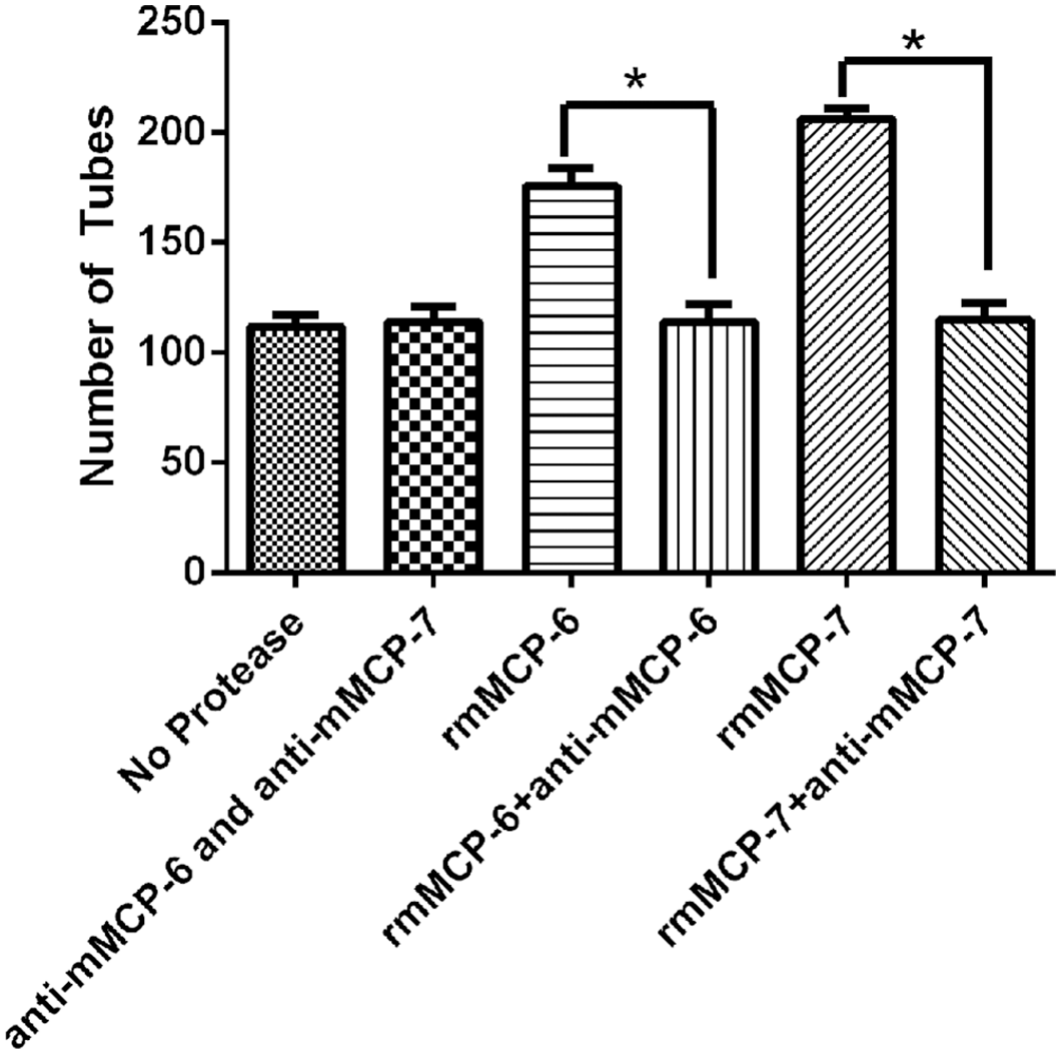
Blocking of tryptases with anti-mMCP-6 and anti-mMCP-7 reduced the number of tubes formed. The proteases were incubated with the respective antibody for 30 min. After pre-incubation the complex (proteases and antibodies) were added and the cells were cultured for 5 hours. Controls cells were cultured for 5 hours in the absence of proteases but in the presence of antibodies. After incubation, tube formation was quantified. Data are presented as mean ± SD from three independent experiments. *p ≤ 0.05.

When the SVEC4-10 cells were cultured with proteases that had been preincubated with specific antibodies (Fig 4), the number of tubes formed by the SVEC4-10 cells was reduced to control levels, confirming the direct action of the proteases on *in vitro* angiogenesis.

### rmMCP-6 and rmMCP-7 induced angiogenesis *in vivo*

**Fig 5:**
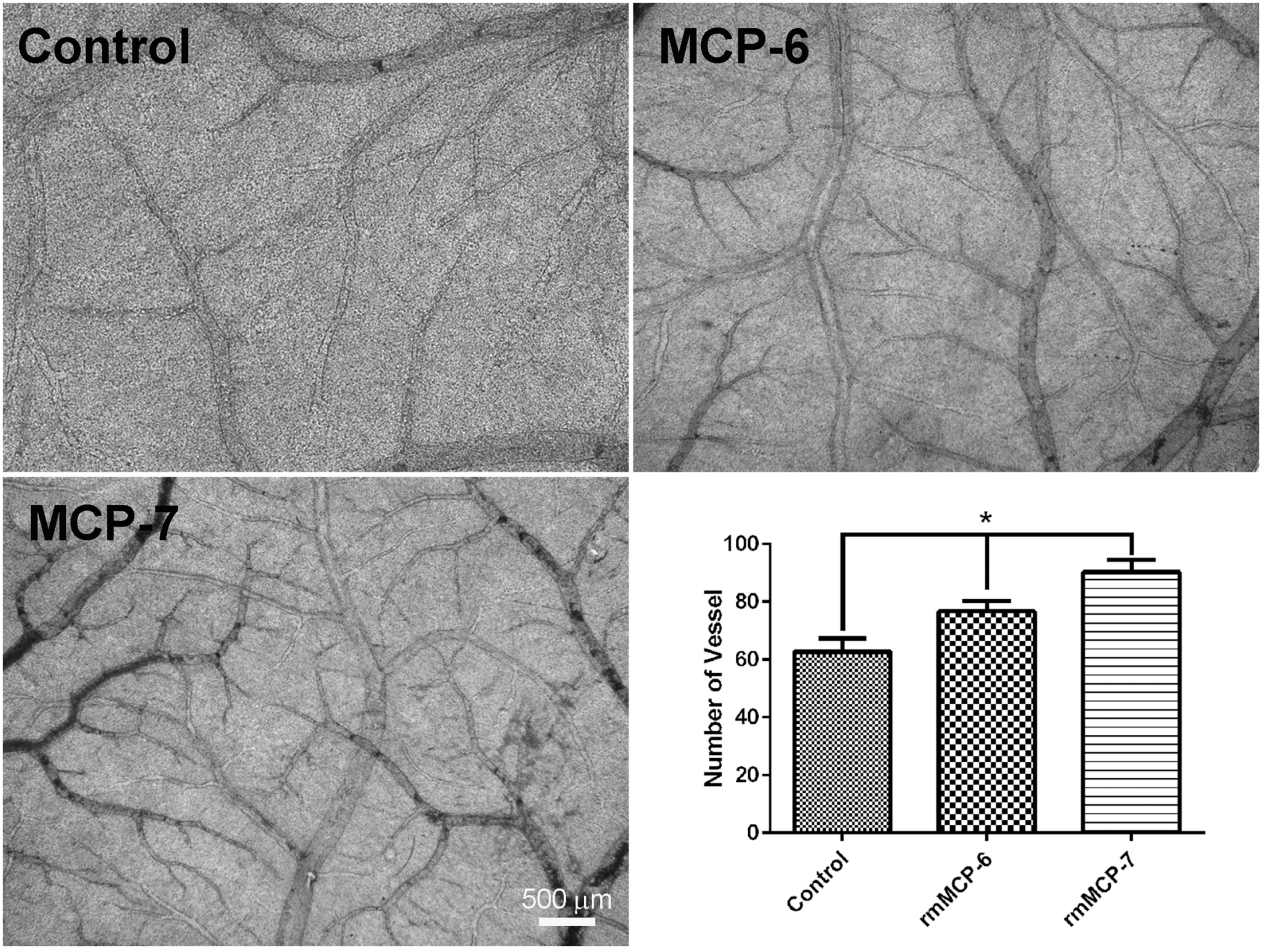
Mast cell tryptases induce an increase of angiogenesis in chicken chorioallantoic membrane. When compared to the control CAM (no tryptases), CAM incubated in presence of rmMCP-6 and 7 showed an increase in the number of blood vessels. Moreover, an increase in blood vessel caliber was also seen in the presence of the tryptases. The graph shows the number of vessel per field (6.5mm^2^) from each sample.

A chick embryo chorioallantoic membrane (CAM) assay was performed to assess the *in vivo* angiogenic ability of rmMCP-6 and 7. The CAM in the presence or absence of rmMCP-6 and 7 was examined on day 12 of incubation in order to analyze angiogenesis. In the presence of rmMCP-6 and 7 an increase in the number and caliber of blood vessels in the CAM was observed (Fig 5) when compared to controls. These results confirm the previous study by Ribatti et al [41] using human mast cell tryptases 6 and 7.

### Mast cell tryptases stimulated invasion of the Geltrex^®^ by SVEC4-10 cells

**Fig 6:**
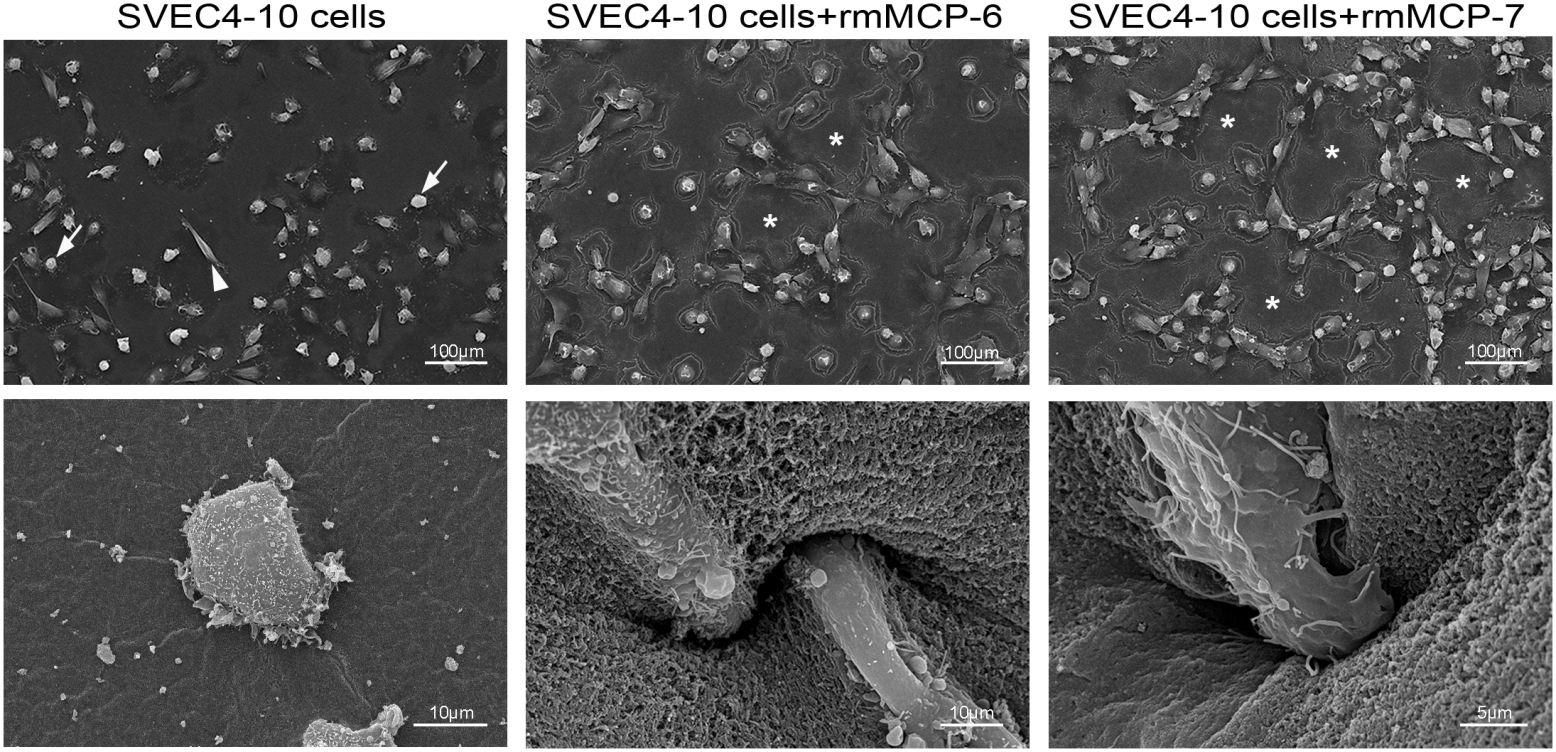
In the presence of rmMCP-6 and rmMCP-7 the endothelial cells invade the Geltrex^®^. SVEC4-10 cells were cultured for 5 hours at 37°C on Geltrex^®^ in the presence of rmMCP-6, rmMCP-7 or in the absence of tryptases. In the absence of rmMCP-6 or -7, most of the SVEC4-10 cells remained rounded (arrows) and few cells spread on the substrate (arrowheads). In the presence of rmMCP-6 or -7 almost all endothelial cells were spread on the substrate and were organized into tubes (asterisks). Additionally, the SVEC4–10 cells invaded the Geltrex^®^ only in the presence of the tryptases.

The effect of rmMCP-6, rmMCP-7 on tube formation was then analyzed by scanning electron microscopy. When cultured for 5 hours on Geltrex^®^, most SVEC4-10 endothelial cells were round, and only a few cells were spread on the substrate (Fig 6). In contrast, when endothelial cells were cultured for 5 hours in the presence of rmMCP-6 or -7, nearly all the cells were fusiform, spread on the substrate and arranged to form loops and tubes. Additionally, the SVEC4–10 cells invaded the Geltrex^®^ in the presence of the tryptases (Fig 6).

### rmMCP-6 and rmMCP-7 did not degrade the Geltrex^®^

**Fig 7:**
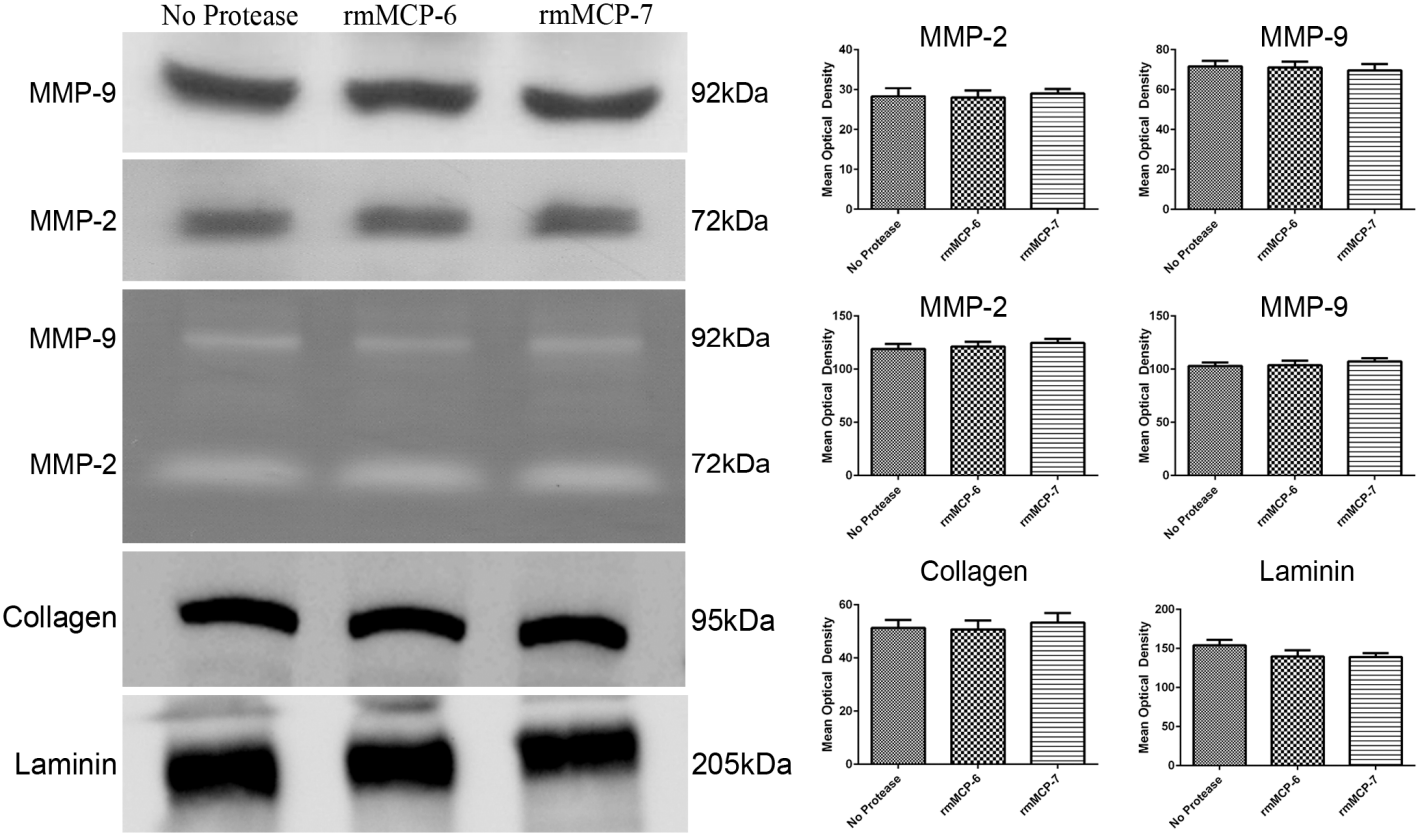
The expression and activity of metalloprotease or gel compound of are not altered in the presence of mast cell tryptases. The cells were cultured for 5 hours at 37°C on Geltrex^®^ in the presence of rmMCP-6, rmMCP-7 or in the absence of tryptases. After incubation, the culture supernatants were used for immunoblotting to analyze metalloprotease expression and for zymograms to determine metalloprotease activity. For gel compounds, after incubation, the Geltrex^®^ was recovered using a lyses buffer and used for immunoblotting to analyze their components. Data are presented as mean ± SD from three independent experiments. *p ≤ 0.05.

The results of the scanning electron microscopy suggested that the Geltrex^®^ may have been partially degraded. To better understand this process, the expression and activity of metalloproteinases (MMP-2 and -9) from the culture supernatant were analyzed. After 5 hours of culture with or without proteases, no changes in expression or activity of metalloproteases were observed (Fig 7). To investigate gel degradation independent of metalloprotease activity, the main components of Geltrex^®^ (laminin and collagen IV) were examined after 5 hours of incubation with or without rmMCP-6 or rmMCP-7. No changes were seen in the amount of laminin or collagen IV in the gel following incubation (Fig 7).

### rmMCP-6 and rmMCP-7 induce the release of angiogenic factors

To investigate whether rmMCP-6 and -7 were able to induce SVEC4–10 cells to release angiogenic factors, the supernatants from the tube formation cultures were analyzed using The Proteome Profiler^™^ Mouse Angiogenesis Array Kit as described in Materials and Methods. The presence of rmMCP-6 induced the release of a total of 40 angiogenic factors and rmMCP-7 stimulated the release of 47 factors. Thirty-six factors were released by both tryptases. Only 4 cytokines, angiogenin, endostatin, IGFBP-3 (Insulin-like Growth Factor-Binding Protein 3) and MCP-1 (Monocyte Chemoattractant Protein-1) were detected in the absence of rmMCP-6 or -7 (Table 1, and S1 Array). Therefore, rmMCP-6 and rmMCP-7 have the ability to stimulate release of angiogenic factors from endothelial cells.

**Table 1:** Angiogenic factors released in the presence of rmMCP-6 or rmMCP-7.

Factor	SVEC4-10	SVEC4-10 + rmMCP-6	SVEC4-10 + rmMCP-7
ADAMTS1	−	−	+
Amphiregulin	−	−	+
Angiogenin	+	+	+
Angiopoietin-1	−	+	+
Angiopoietin-3	−	+	+
Coag. Factor III	−	+	+
CXCL16	−	+	+
Cyr61	−	−	+
DLL4	−	−	+
DPPIV	−	+	+
Endoglin	−	+	+
Endostatin	+	+	+
Endothelin-1	−	+	+
FGF acidic	−	+	+
FGF basic	−	−	+
KGF	−	−	+
Fractalkine	−	−	+
GM-CSF	−	+	−
HB-EGF	−	+	+
HGF	−	+	+
IGFBP-1	−	+	+
IGFBP-2	−	+	+
IGFBP-3	+	+	+
IL-1α	−	+	+
IL-10	−	−	+
IP-10	−	−	+
KC	−	+	+
Leptin	−	+	+
MCP-1	+	+	+
MIP-1α	−	+	+
MMP-3	−	+	+
MMP-8	−	+	+
MMP-9	−	+	+
NOV/ IGFBP-9	−	−	+
Osteopontin	−	+	+
PD-ECGF	−	+	+
PDGF-AA	−	+	+
PDGF-AB	−	+	+
Pentraxin-3	−	+	+
Platelet Factor 4	−	+	+
PlGF-2	−	+	+
Prolactin	−	+	+
Proliferin	−	−	+
SDF-1	−	+	−
Serpin E1	−	+	+
Serpin F1	−	+	−
Thrombospondin-2	−	+	−
TIMP-1	−	+	+
TIMP-4	−	+	+
VEGF	−	+	+
VEGF-B	−	+	+

The Proteome Profiler^™^ Mouse Angiogenesis Array Kit was used to simultaneously assess the relative levels of mouse angiogenesis-related proteins of the supernatant from the tube formation assay in the presence or absence of rmMCP-6 and rmMCP -7.

### rmMCP-6 and rmMCP-7 induced the differential release of angiogenic factors

**Fig 8:**
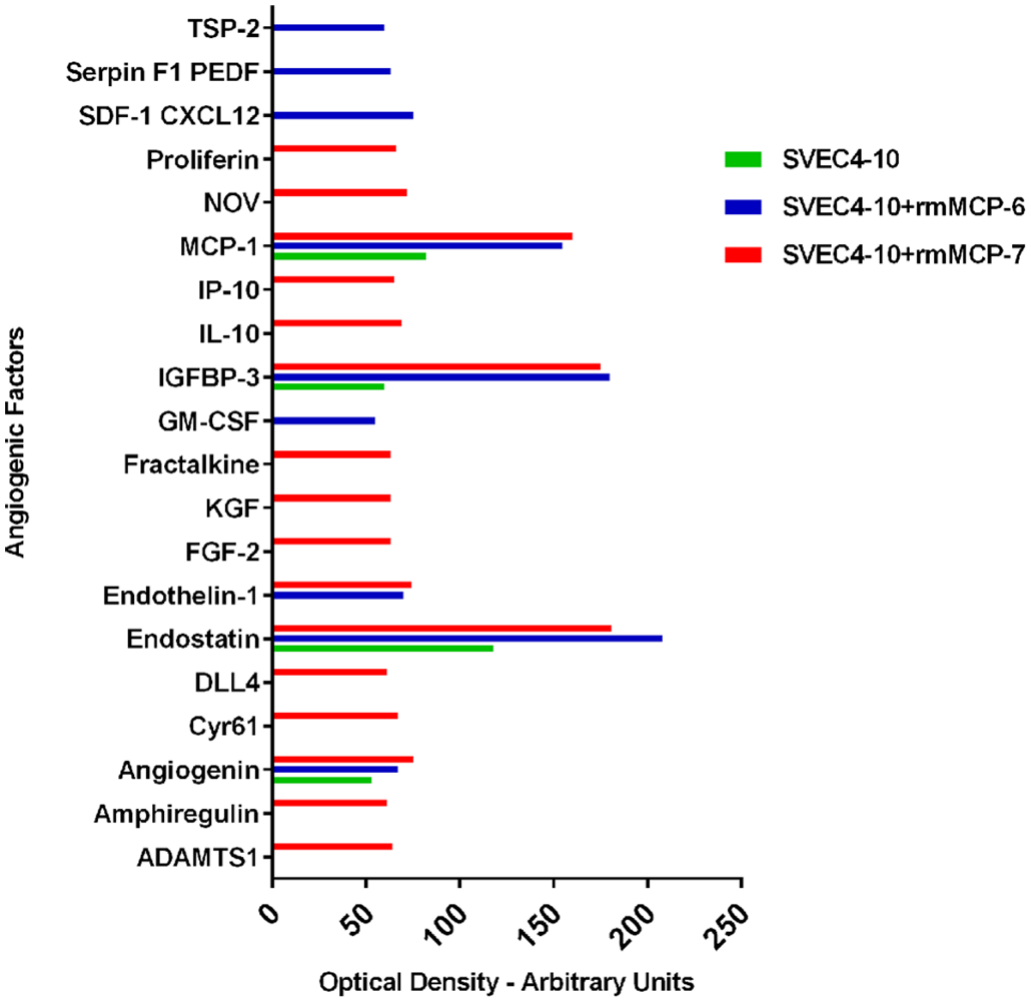
In the presence of rmMCP-6 and rmMCP-7 SVEC4-10 cells show a differential release of angiogenesis related cytokines/proteins. The graph shows the cytokines/proteins that were released exclusively by rmMCP-6 (blue) or rmMCP-7 (red). Four cytokines/proteins that were released by control cells were increased in the presence of the tryptases (green). The cells were cultured for 5 hours at 37°C on Geltrex^®^ in the presence of rmMCP-6, rmMCP-7 or in the absence of tryptases. After incubation, a mouse cytokine array kit was used to analyze the protein expression of different pro- and anti-angiogenic factors in the culture supernatants. The data shown is the mean spot pixel density that was quantified from the arrays using the image analysis software Adobe Photoshop CS6 V 13.0.

The profile of the factors released after incubation with rmMCP-6 was different from that seen with rmMCP-7. Four factors released by control SVEC4-10 cells were significantly increased in the presence of the tryptases (Fig 8 and S1 Array). This difference may be a reflection of their physiological functions (Table 2).

**Table 2:** Action of differentially released angiogenic factors.

Factors released by SVEC4-10 cells		
Angiogenic Fator	Role in Angiogenesis	Reference
Angiogenin	Induces the proliferation and migration of endothelial cells	[42, 43]
Endostatin	Inhibits endothelial cells proliferation, migration and angiogenesis	[44, 45]
IGFBP-3	Promotes angiogenesis and cell motility	[42]
MCP-1	Induces migration, sprouting of endothelial cells, and induces vascular-like tunnel formation *in vivo*	[46, 47]
**Factors Released Exclusively in the Presence of rmMCP-6**		
GM-CSF	Induces proliferation and migration of endothelial cells	[42]
SDF-/CXCL12	Induces the activation of matrix metalloproteinase-9 (MMP-9)	[48]
Serpin F1	Stimulates VEGF secretion, activates integrins, interferes with VEGF signaling thereby inhibiting angiogenesis	[49, 50]
Thrombospondin -2	Inhibits endothelial cell function	[51]
**Factors Released Exclusively in the Presence of rmMCP-7**		
ADAMTS1	Induces degradation of venular basement membrane versican in VEGF-induced pathological angiogenesis	[52, 53]
Amphiregulin	Induces the proliferation of rat vascular smooth muscle cells, possibly implicated in arterial remodeling	[54, 55]
Cyr61	Induces angiogenesis *in vivo*, supports cell adhesion, promotes cell migration, and enhances growth factor-stimulated mitogenesis in endothelial cells.	[56, 57]
Dll4	Regulates the specification of endothelial cells into tip and stalk cells during angiogenic sprouting	[58, 59]
FGFb	Regulates proliferation, migration and differentiation of endothelial cells	[60]
KGF	Directly stimulates capillary endothelial cells’ proliferation and migration	[61, 62]
Fractalkine	Stimulates *ex vivo* and *in vivo* angiogenesis, may act as a direct angiogenic modulator in endothelial cells without inducing VEGF expression	[63]
NOV/IGFBP-9	Acts directly upon endothelial cells to stimulate pro-angiogenic activities, and induces angiogenesis *in vivo*	[64, 65]
IL-10	Promotes pathological angiogenesis and interferes with endothelial differentiation	[66, 67]
IP-10	Potent inhibitor of angiogenesis *in vivo*	[68]
Proliferin	Stimulates endothelial cell migration, invasion, and tube formation *in vitro*, and angiogenesis *in vivo*	[69]

Biological function of angiogenic factors shown in Fig 8.

## Discussion

The results of this study show that the tryptases, rmMCP-6 and -7, play an important role in angiogenesis, and can act directly on endothelial cells independently of other mediators released by mast cells. Furthermore, rmMCP-7 is more effective in accelerating this process.

During tube formation, the endothelial cells aggregated faster in the presence of rmMCP-7, thus suggesting that rmMCP-7 may induce or accelerate the anastomosis of endothelial cells during angiogenesis. In contrast, the mean area occupied by the loops was increased when the endothelial cells were cultured with rmMCP-6. The area occupied by the loops is directly related to vessel diameter, thus suggesting that mMCP-6 can influence the diameter of the vessels during angiogenesis. The diameter of new capillary sprouts can be influenced by the local concentration and the action of angiogenic factors on endothelial cells [70]. Angiogenic factors may also play a role in determining lumen diameter, as well as vessel length during angiogenesis [71, 72]. The fact that rmMCP-6 and 7 also induced endothelial cells to invade the Geltrex^®^, suggests that these proteases stimulate endothelial cells to migrate contributing to the formation of new blood vessels. This phenomenon was not observed when the cells are cultured without proteases.

No changes were seen in the amount of laminin or collagen IV in the gel following incubation, however, the results of the scanning electron microscopy showed that the endothelial cells invaded the Geltrex^®^. These findings suggest that the invasion occurs independently of matrix degradation. However, a small amount of degradation may be occurring that is not detected by Western blot. Invasion independent of degradation was also observed by other investigators. Wolf and colleagues [73] demonstrated that amoeboid migration appears to use morphodynamic supramolecular mechanisms to bypass tissue barriers independent of ECM degradation. Furthermore, cortactin and cofilin appear to regulate invadopodial elongation in MDA-MB-231 cells independent of degradation [74].

Angiogenesis array analysis showed that there was a differential release of cytokines/angiogenic proteins during the *in vitro* angiogenesis. In the absence of tryptases only 4 factors were released from the SVEC10-4 cells: angiogenin, endostatin, IGFBP-3, MCP-1. Angiogenin induces the proliferation and migration of endothelial cells [42]. Endostatin down-regulates signaling pathways associated with proangiogenic activity and upregulates many antiangiogenic genes [75]. IGFBP-3 promotes angiogenesis and cell motility [42, 76, 77] while MCP- 1 induces migration and sprouting of endothelial cells, besides induced vascular-like tunnel formation in vivo [46, 47].

The concentration of most cytokines released into the supernatant of SVEC4-10 endothelial cells cultured with either rmMCP-6 or -7 were similar. However, some differences were observed when endothelial cells were cultured with rmMCP-6 or rmMCP-7. In the presence of rmMCP-6, the endothelial cells released GM-CSF, SDF-1, Serpin F1, and Trombopondin-2. These cytokines are directly related to cell proliferation and migration [42]. rmMCP-7 induced the release of cytokines that are considered to be extremely potent angiogenesis inducers such as, ADAMTS1, Amphiregulin, Cyr61, Dll4, FGFb, KGF, Fractalkine, NOV/IGFBP-9, IL-10, IP-10 (Interferon gamma-induced protein 10), and proliferin [78, 79]. Interestingly, rmMCP-7 induced the release of Dll4, which is responsible for the differentiation of endothelial cells into tip or stalk cells via the Notch signaling pathway [80–83]. The Notch pathway controls both normal and pathological angiogenesis by modulating the development of tip cells and stalk cells during the formation of new blood vessels [84]. The release of this cytokine may explain the increased spreading of endothelial cells when rmMCP-7 was present during the tube formation assays.

Several previous studies have shown that tryptase can induce migration and proliferation of endothelial cells [32, 85, 86]. Endothelial cell proliferation induced by tryptase can be in directly attributed to its proteolytic activity on PAR-2 [87, 88]. Tryptase can also indirectly affect angiogenesis by stimulating the α1(I) procollagen synthesis by fibroblast and activating dermal fibroblasts [89]. Ribatti et al. [41] showed that human tryptases stimulate angiogenesis in CAM, similar to what was observed in the present study. The results of both studies confirm the angiogenic activity of these proteases and indicate that tryptase interacts with endothelial cells via unidentified mechanisms to induce angiogenesis.

Taken together, the results of the present study show the extensive angiogenic activity of tryptases and demonstrate that the tryptase subtypes (rmMCP-6 and-7) have different roles during *in vitro* angiogenesis. Both proteases induced cell migration and adhesion, however, rmMCP-7 also stimulated and accelerated the anastomosis of endothelial cells during the angiogenesis process. Other studies have shown distinct functions for tryptase subtypes in inflammation [30, 90–92].

Understanding the specific role of each tryptase subtype in angiogenesis can be of great importance in developing new therapeutic interventions, or in the improvement of existing ones, that aim to inhibit the formation of blood vessels in pathological processes.

## Supporting Information

S1 Array Results of the angiogenesis protein array. The cells were cultured for 5 hours at 37°C on Geltrex^®^ in the presence of rmMCP-6, rmMCP-7 or in the absence of tryptases. After incubation, The Proteome Profiler^™^ Mouse Angiogenesis Array Kit was used to analyze the protein expression of different pro- and anti-angiogenic factors in culture supernatants. Array membrane images are shown. The table gives the mouse angiogenesis array coordinates with a description, location and the mean spot pixel density of each angiogenic factor in the membrane array. The mean spot pixel density was quantified from the arrays using image analysis software Adobe Photoshop CS6 V 13.0.
(TIF)

S1 Fig Original immunoblot and zymography data presented in this study. Red outlines represent the immunoblot sections presented in Fig 6.
(TIF)
