# Supplementary figures and images for: Mast Cell Proteases 6 and 7 Stimulate Angiogenesis by Inducing Endothelial Cells to Release Angiogenic Factors
Source: PLoS One. 2015 Dec 3;10(12):e0144081. doi: 10.1371/journal.pone.0144081 (PMC4669151; doi:10.1371/journal.pone.0144081)

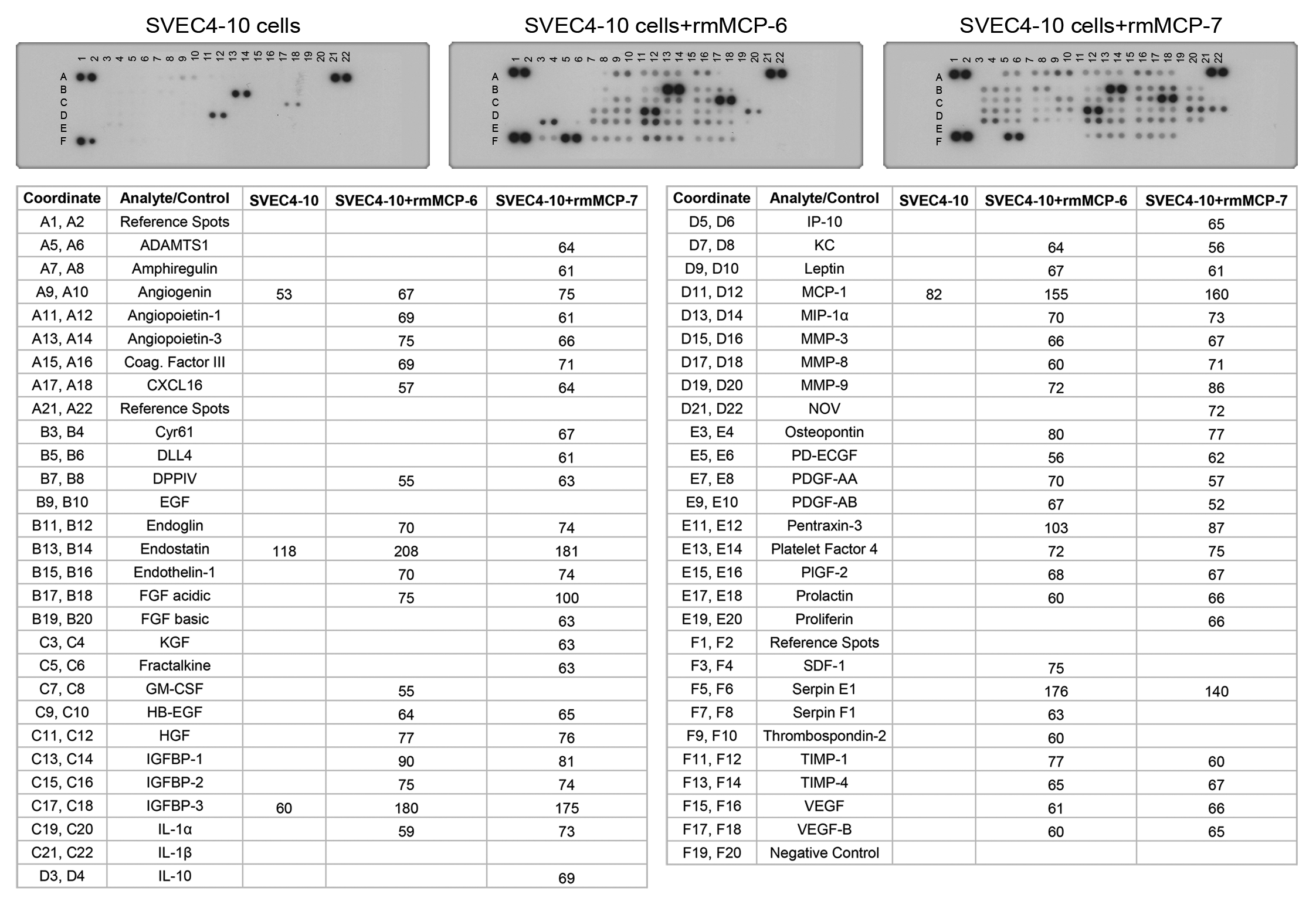

Supplement: S1 Array — The cells were cultured for 5 hours at 37°C on Geltrex® in the presence of rmMCP-6, rmMCP-7 or in the absence of tryptases. After incubation, The Proteome Profiler™ Mouse Angiogenesis Array Kit was used to analyze the protein expression of different pro- and anti-angiogenic factors in culture supernatants. Array membrane images are shown. The table gives the mouse angiogenesis array coordinates with a description, location and the mean spot pixel density of each angiogenic factor in the membrane array. The mean spot pixel density was quantified from the arrays using image analysis software Adobe Photoshop CS6 V 13.0. (TIF) [file pone.0144081.s001.tif]

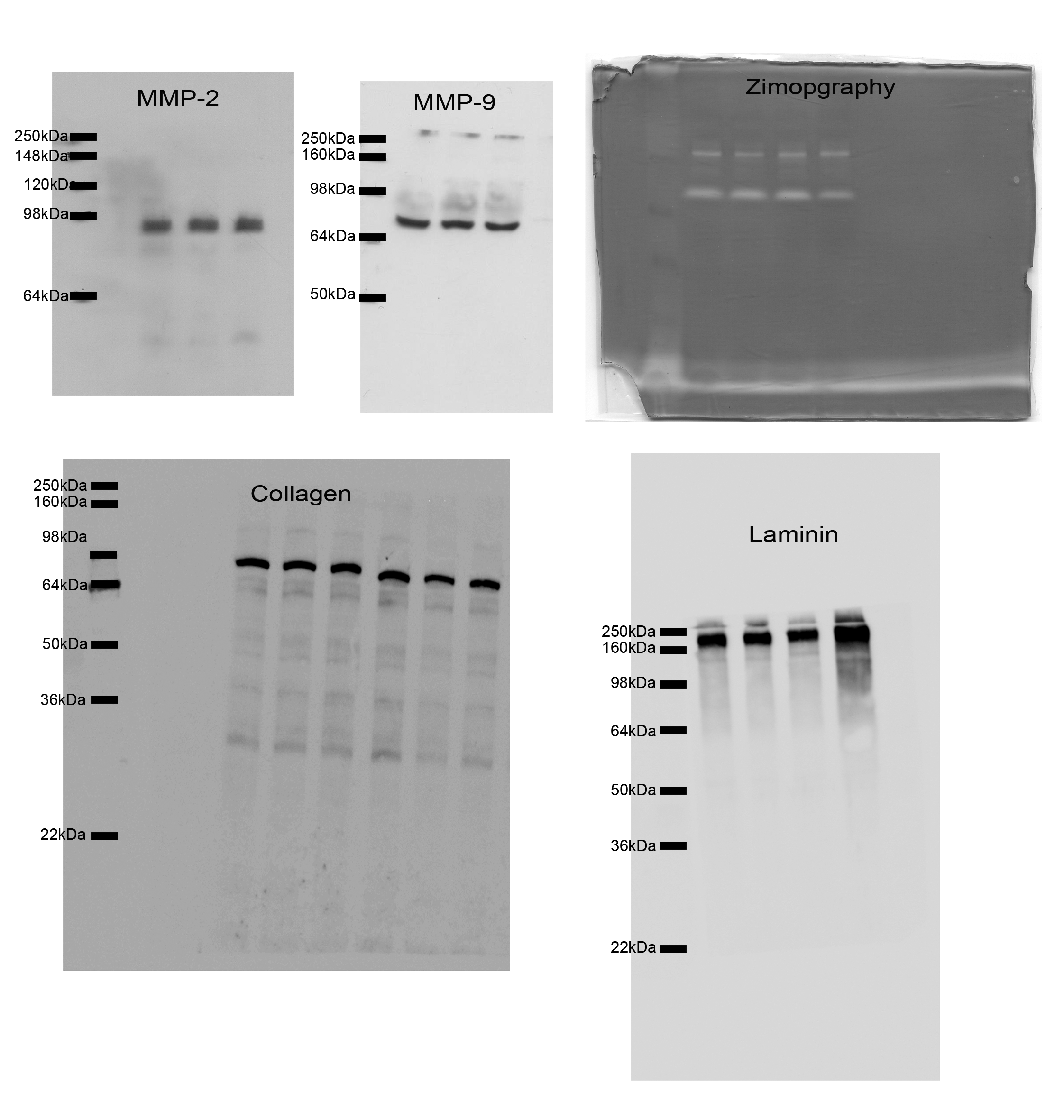

Supplement: S1 Fig — Red outlines represent the immunoblot sections presented in Fig 6. (TIF) [file pone.0144081.s002.tif]
